# Acute Effects of Low-Intensity Electrical Stimulation on Segmental Arterial Stiffness

**DOI:** 10.3389/fphys.2022.828670

**Published:** 2022-06-06

**Authors:** Hiroyuki Oda, Mami Fujibayashi, Naoyuki Matsumoto, Masato Nishiwaki

**Affiliations:** ^1^ Faculty of Health Science, Morinomiya University of Medical Science, Osaka, Japan; ^2^ Faculty of Engineering, Osaka Institute of Technology, Osaka, Japan; ^3^ Faculty of Agriculture, Setsunan University, Osaka, Japan; ^4^ Faculty of Environmental Symbiotic Science, Prefectural University of Kumamoto, Kumamoto, Japan

**Keywords:** health, pulse wave velocity, passive, arteriosclerosis, cardio-ankle vascular index (CAVI)

## Abstract

Electrical muscle stimulation (EMS) has traditionally been employed to improve muscle strength and glucose uptake. EMS may also reduce arterial stiffness, but little is known about whether low-intensity EMS reduces systemic and/or regional arterial stiffness. This study aimed to examine the effects of low-intensity EMS of the lower limbs on segmental arterial stiffness. Fourteen healthy subjects participated in experiments under two different protocols (control resting trial (CT) and electrical stimulation trial (ET)) in random order on separate days. The EMS was applied to the lower limbs at 4 Hz for 20 min at an intensity corresponding to an elevation of approximately 15 beats/min in pulse rate (10.7 ± 4.7% of heart rate reserve). Arterial stiffness was assessed by cardio-ankle vascular index (CAVI), CAVI_0_, heart-ankle pulse wave velocity (haPWV), brachial-ankle pulse wave velocity (baPWV), heart-brachial pulse wave velocity (hbPWV), and carotid-femoral pulse wave velocity (cfPWV). In both trials, each parameter was measured at before (Pre) and 5 min (Post 1) and 30 min (Post 2) after trial. After the experiment, CT did not cause significant changes in any arterial stiffness parameters, whereas ET significantly reduced CAVI (from Pre to Post 1: −0.8 ± 0.5 unit *p* < 0.01), CAVI_0_ (from Pre to Post 1: −1.2 ± 0.8 unit *p* < 0.01), haPWV (from Pre to Post 1: −47 ± 35 cm/s *p* < 0.01), and baPWV (from Pre to Post 1: −120 ± 63 cm/s *p* < 0.01), but not hbPWV or cfPWV. Arm diastolic blood pressure (BP) at Post 2 was slightly but significantly increased in the CT compared to Pre or Post 1, but not in the ET. Conversely, ankle diastolic and mean BPs at Post 1 were significantly reduced compared to Pre and Post 2 in the ET (*p* < 0.01). These findings suggest that low-intensity EMS of the lower limbs reduces arterial stiffness, but only in sites that received EMS.

## Introduction

Arterial stiffness has been identified as an independent risk factor for future cardiovascular disease (Laurent and Boutouyrie, 2007). Pulse wave velocity (PWV) and cardio-ankle vascular index (CAVI) are well established indices of arterial stiffness (Vlachopoulos et al., 2010; Shirai et al., 2011). Various reports have indicated that PWV increases with age (Avolio et al., 1985), and large elastic arteries progressively stiffen with age, even in healthy individuals (Avolio et al., 1983; Gando et al., 2010). Arterial stiffening impairs the ability of arteries to buffer the pulsations of blood pressure (BP) and flow (Tanaka, 2019), so preventing arterial stiffening is of paramount importance. Further scientific evidence of simple and effective methods for preventing arterial stiffening is therefore needed.

Exercises, particularly aerobic exercise, are widely known to be important for reducing arterial stiffness. Indeed, cross-sectional studies have shown that arterial stiffness is lower in runners, cyclists, and swimmers than in control subjects (Otsuki et al., 2007; Nishiwaki et al., 2017). Acute and regular aerobic exercise interventions such as walking, running, cycling, and swimming have also gained acceptance as ways to reduce arterial stiffness (Kingwell et al., 1997; Tanaka et al., 2000; Wang et al., 2014; Muller et al., 2015; Zhou et al., 2015; Wong et al., 2018). Although such aerobic exercises would improve arterial stiffness (Tanaka, 2019), severe obesity and painful lower limb osteoarthritis (OA) may present obstacles to regular aerobic exercise. Providing effective passive approaches for individuals with exercise restrictions could be valuable for reducing the risk of cardiovascular disease. In recent years, electrical muscle stimulation (EMS) has been gaining attention as a means of addressing such problems. EMS induces passive muscle activity, and is well known to improve glucose metabolism and aerobic capacity (Hamada et al., 2003; Hamada et al., 2004; Miyamoto et al., 2016). Some human and animal studies have also reported that EMS can increase blood flow and shear stress, and these circulatory alterations could in turn induce a reduction in arterial stiffness (Hudlicka and Price, 1990; Hang et al., 1995; Annex et al., 1998; Hudlicka, 1998; Janssen and Hopman, 2003). In fact, regular EMS intervention reduces arterial stiffness in patients with chronic heart failure and pulmonary arterial hypertension (Dobsak et al., 2012; Kahraman et al., 2020). Applying the passive approach such as EMS could thus improve arterial stiffness similar to voluntary aerobic exercise. However, previous studies performed relatively high-intensity and resistance exercise (10–80 Hz)-simulated EMS (Dobsak et al., 2012; Kahraman et al., 2020). High-intensity EMS generally induces pain, discomfort, and muscle fatigue (Muro et al., 1986). Therefore, in the future, to provide intervention programs for rehabilitation of chronic heart failure or pulmonary arterial hypertension patients and to prevent cardiovascular diseases, it is important to clarify the effect of low-intensity EMS on the arterial stiffness. In addition, to confirm the safety of intervention programs using low-intensity EMS, initial studies on healthy individuals are required.

In the case of passively simulating aerobic exercise, EMS is widely applied to the lower skeletal muscle at relatively low intensity, because the lower limb contains many large muscles and low-intensity stimuli can reduce pain, discomfort, and risks of injury to the joints or muscles. However, one-legged exercises at low-intensity reduce arterial stiffness in the exercised leg, but not in the non-exercised leg or central part, and alterations in arterial stiffness may be predominately mediated by local factors rather than systemic factors (Sugawara et al., 2003; Heffernan et al., 2006; Yamato et al., 2021). Although local and systemic arterial stiffness are important, arterial stiffness of the central region (i.e., aortic arterial stiffness) has generally been proposed as the reference standard. Thus, whether low-intensity EMS of the lower limbs can actually reduce stiffness of the central and systemic arteries (including in the lower limbs) is an important point that remains unclear. The effects of low-intensity EMS of the lower limbs on segmental arterial stiffness thus remain controversial. Such studies can offer important new insights into the development of effective simulated exercise methods to reduce arterial stiffness and also contribute to a better understanding of the physiological mechanisms involved. To the best of our knowledge, however, no data are available regarding EMS or segmental arterial stiffness.

Based on this background, we hypothesized that low-intensity EMS of the lower limbs would induce reductions in arterial stiffness parameters involving the lower limbs, but not in body parts that did not receive EMS. The present study thus aimed to examine the effects of low-intensity EMS on segmental arterial stiffness.

## Materials and Methods

### Subjects

Fourteen male Japanese college students (age range, 18–22 years) at the Osaka Institute of Technology participated in this study. Only males were recruited for this study because the student cohort at the institution is predominantly male and there were no female responders to local advertisements or referrals. Mean age, height, body mass, and body mass index (BMI) were 20 ± 2 years, 170.7 ± 3.5 cm, 64.6 ± 9.5 kg, and 22.2 ± 3.5 kg/m^2^, respectively. All participants were non-smokers and were not taking any medications or supplements at the time of enrolment. Patients were not obese (BMI <30 kg/m^2^) and did not have hypertension (systolic BP < 140 mmHg and diastolic BP < 90 mmHg). None had any history of chronic diseases that could affect cardiovascular health, metabolism, or exercise. Participants had not participated in any regular exercise programs, such as club, team, or extracurricular activities of sports, for at least the previous 2 years. The purpose, procedures, and risks of the study were explained to all participants and written informed consent was obtained prior to enrolment in the study. All study protocols were reviewed and approved by the Human Ethics Committee at Osaka Institute of Technology (approval number: 2016-12) and were conducted in accordance with the guidelines of the Declaration of Helsinki.

### Sample Size and Experimental Procedures

We determined the appropriate and minimum sample sizes before the study by calculations using G*Power version 3.1. We assumed that CAVI would change by 5–10% based on our previous other findings (Nishiwaki et al., 2015; Nishiwaki et al., 2020). A sample size of at least 22 participants in total (11 samples per trial) was needed to detect an effect size (ES) (f) of 0.25 (medium) at 80% power with an α of 5% using a within-between interaction of two-way repeated-measures analysis of variance (ANOVA). We further assumed that changes in arterial stiffness would be transiently shown by a difference of ≥5–7% (ES (dz), 1.0) in a pretest-posttest design according to previous findings (Kingwell et al., 1997; Wang et al., 2014; Yamato et al., 2016). At least 10 subjects were needed to detect this difference at 80% power with a two-tailed α of 5%. We therefore planned to recruit 14 subjects (total sample size, 28) to account for any sudden cancellations or withdrawals from the study.

**FIGURE 1:**
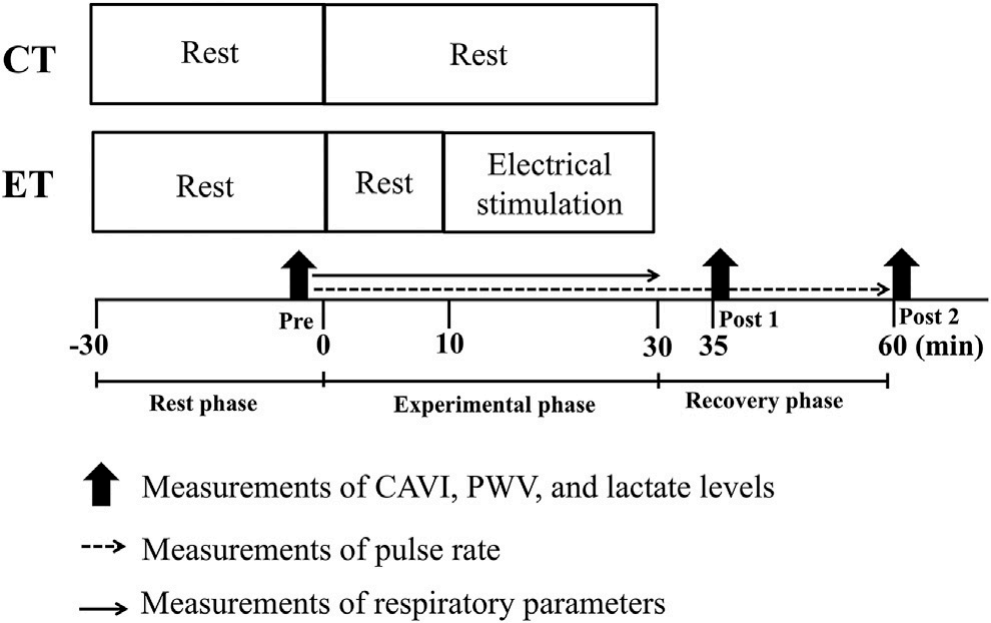
Time course of the experimental protocol. CT, control resting trial; ET, electrical stimulation trial; Pre, pre-experiment; Post 1, 5 min post-experiment; Post 2, 30 min post-experiment; CAVI, cardio-ankle vascular index; PWV, pulse wave velocity. All subjects performed each condition in random order on different days.

All experiments were conducted in a quiet, air-conditioned room at 24–25°C. To avoid potential diurnal variations, two experiments for each participant were performed at the same time of day at least 4 h after a light meal. The range of start times for each trial was 10:00–17:00. All subjects abstained from vigorous exercise for at least 24 h before testing, and from caffeine and food for 4 h before testing. In addition, participants were advised to eat their usual habitual breakfast, lunch, or dinner the day before each experiment, and similar standard contents and mealtimes without irregularity within and between participants were confirmed from checklist questionnaires and face-to-face interview when subjects attended our laboratory. Figure 1 shows the time course of the study day. All subjects were assigned in random sequence to one trial a day for 2 days. Trials comprised a control resting trial (CT) and an electrical stimulation trial (ET). Each trial was performed approximately 1 week apart, and the mean time between test sessions was 5 ± 1 days.

In both trials, all subjects arrived at the laboratory and rested for at least 30 min, then PWVs, hemodynamic parameters, and blood lactate concentrations were assessed in the supine position to establish pre-trial baselines (Rest phase). In the ET, after sitting for 10 min, participants underwent 20 min of EMS of lower limbs using an electrical stimulator (B-SES SL1; Homer Ion Japan, Tokyo, Japan) in the sitting position on a bed, as previously described (Miyamoto et al., 2016). Three rubber stimulation surface electrodes were placed over the lower thigh (proximally and distally) and ankle. Stimulator output was selected as metabolism mode (4 Hz), and stimulation intensity was set to create a 15 ± 5 beats/min elevation in pulse rate (PR), corresponding to approximately 10.7 ± 4.7% of heart rate reserve (HRR) and low-intensity aerobic exercise (Kume et al., 2021). High frequency electrical stimulation is likely to induce tetanic muscle activity and cause muscle fatigue (Muro et al., 1986). Thus, we used 4 Hz stimulation frequency in this study. And also, electrocardiographic signals and heart rate could not be appropriately recorded during EMS due to interference from the EMS, we monitored PR using a finger clip sensor for a relative intensity setting. Thus, the stimulation intensity (i.e., degrees of effective electrical current) in the present study was manually adjusted according to the PR level of each subject. On the other hand, subjects in the CT rested on the same bed as ET for 30 min. In addition to PR, respiratory gas parameters were continuously monitored during both trials. After 30 min of an experimental phase (i.e., CT or ET), each subject rested in a comfortable chair for 30 min while biometric measurements were repeated at 5 min (Post 1) and 30 min (Post 2) in the post-experimental phase as a recovery phase.

### Assessment of Each Parameter

The same investigators measured all parameters. Throughout the study period from rest phase to recovery phase, PR was monitored using a finger clip monitor (SAT-2200; Nihon Kohden, Tokyo, Japan). During each trial (i.e., in the experimental phase), oxygen uptake [ ${\text{V}}^{・}$ O_2_], carbon dioxide output [ ${\text{V}}^{・}$ CO_2_], and expired ventilation [ ${\text{V}}^{・}$ E], respiratory exchange ratio (RER) was determined every 15 s using an automatic gas analyzer with a mixing chamber. Specifically, expired gas volume was measured using the flow sensor of a Respiromonitor (RM-300; Minato Medical Science, Tokyo, Japan) and oxygen and carbon dioxide concentrations were measured using a portable gas monitor (AR-10; Arco System, Chiba, Japan) that was calibrated and confirmed before each test according to the instructions from the manufacturer. Each respiratory gas parameter was then automatically calculated using analysis software (AT Windows; Minato Medical Science, Osaka, Japan), and averaged every 5 min. In addition, approximate metabolic equivalents (METs) were calculated using the following equation: METs = ${\text{V}}^{・}$ O_2_ at 30 min/ ${\text{V}}^{・}$ O_2_ at baseline. Day-to-day coefficient of variation (CV) for ${\text{V}}^{・}$ O_2_ during the same exercise was 5.3 ± 1.5%, as determined in eight individuals on two separate days (Nishiwaki et al., 2017). Before and 5 and 30 min after each trial, lactate levels were also measured using a lactate analyzer (Lactate Pro 2; Arklay, Kyoto, Japan).

BP and PWVs were measured with the patient supine using a VS-1500AE/AN semi-automated device (Fukuda Denshi, Tokyo, Japan) as assessment parameters for each trial before and 5 and 30 min after each trial (Nishiwaki et al., 2017; Nishiwaki et al., 2019; Nishiwaki et al., 2020; Ogawa et al., 2020). Cuffs to measure BP and PWV were wrapped around both upper arms and the ankles, then CAVI, heart-ankle PWV (haPWV), brachial-ankle PWV (baPWV), and heart-brachial PWV (hbPWV) were used as indices of arterial stiffness. CAVI was automatically calculated from five pulse-wave signals and arm BP using the following formula (Shirai et al., 2011; Nishiwaki et al., 2014) (Eq. 1).

$$\text{CAVI}=\text{a}\left[\left(\frac{2\text{ρ}}{\text{PP}}\right)\cdot In\;\left(\frac{SBP}{DBP}\right)\cdot PWV^{2}\right]+b$$ (1)

The values of *a* and *b* are constants. *ρ* is the blood density. *PP* is SBP-DBP. *SBP* and DBP are arm systolic and diastolic BPs, respectively.

Carotid-femoral PWV (cfPWV), as an index of central arterial stiffness, was measured using the same device in another measurement mode. Carotid and femoral arterial pressure waveforms were recorded by amorphous pulse wave sensors (TY-501A; Fukuda Denshi, Tokyo, Japan) attached to the carotid and femoral arteries, and values were automatically calculated as the distance between the carotid and femoral artery sites divided by transit time. The CVs for CAVI, haPWV, baPWV, hbPWV, and cfPWV measurements on two separate days (reproducibility) were 3.6 ± 0.6%, 2.6 ± 0.6%, 2.7 ± 0.3%, 4.2 ± 0.6%, and 7.5 ± 1.2%, respectively (Nishiwaki et al., 2014; Nishiwaki et al., 2015). Recent studies have reported that CAVI_0_ is similar index to CAVI and is theoretically more independent of BP. The CAVI and arm systolic and diastolic BPs were then used to calculate CAVI_0_ (Spronck et al., 2017; Giudici et al., 2021) (Eq. 2).

$$CAVI_{0}=\frac{CAVI-b}{a}\cdot \frac{\frac{P_{s}}{P_{d}}-1}{ln\left(\frac{P_{s}}{P_{d}}\right)}-ln\left(\frac{P_{d}}{P_{ref}}\right)$$ (2)

The values of *a* and *b* in the CAVI equation are different for different ranges of CAVI. *P*_*ref*_ = 100 mmHg as a reference pressure. *Ps* and *Pd* is right arm systolic and diastolic BP, respectively.

### Statistical Analysis

All values are presented as mean and standard deviation (SD). Changes in each parameter were analyzed by two-way (trial × time) repeated-measures ANOVA. When the F-value was significant, Bonferroni correction was applied for post-hoc multiple comparisons. Changes in arterial stiffness between Pre and Post 1 trial were performed using the paired *t*-test. All data were statistically analyzed using Sigma Stat version 2.03 (Systat Software Inc., San Jose, CA, United States). To quantify the magnitude of the experimental effect between Pre and Post 1, ES (dz) was calculated using G*Power version 3.1. A criterion alpha level of *p* < 0.05 was used to determine statistical significance for all data.

## Result

### Changes in Physiological Parameters During the CT and ET

**FIGURE 2:**
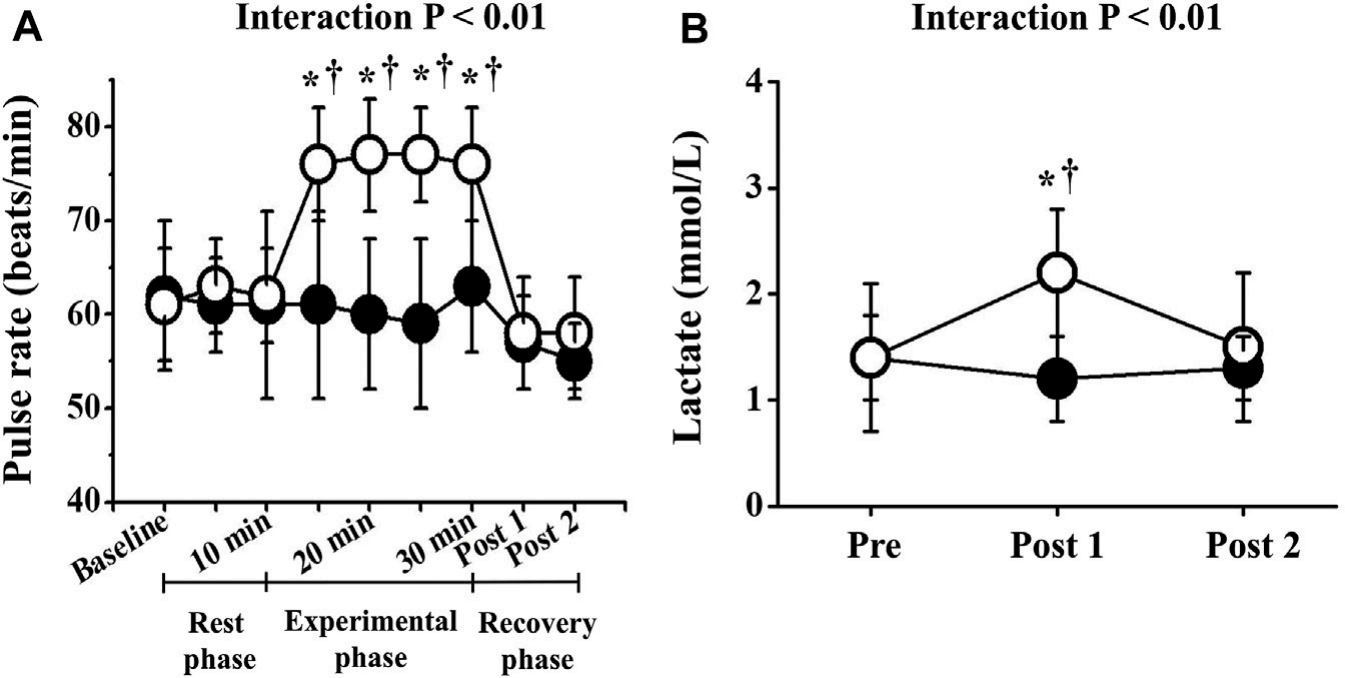
Temporal changes in pulse rate **(A)** and blood lactate **(B)**. Filled circles (•), control resting trial; open circles (○), electrical stimulation trial. Other abbreviations are the same as in Figure 1. ∗*p* < 0.01 vs. CT; †*p* < 0.01 vs. baseline. Data are given as mean ± SD.

**FIGURE 3:**
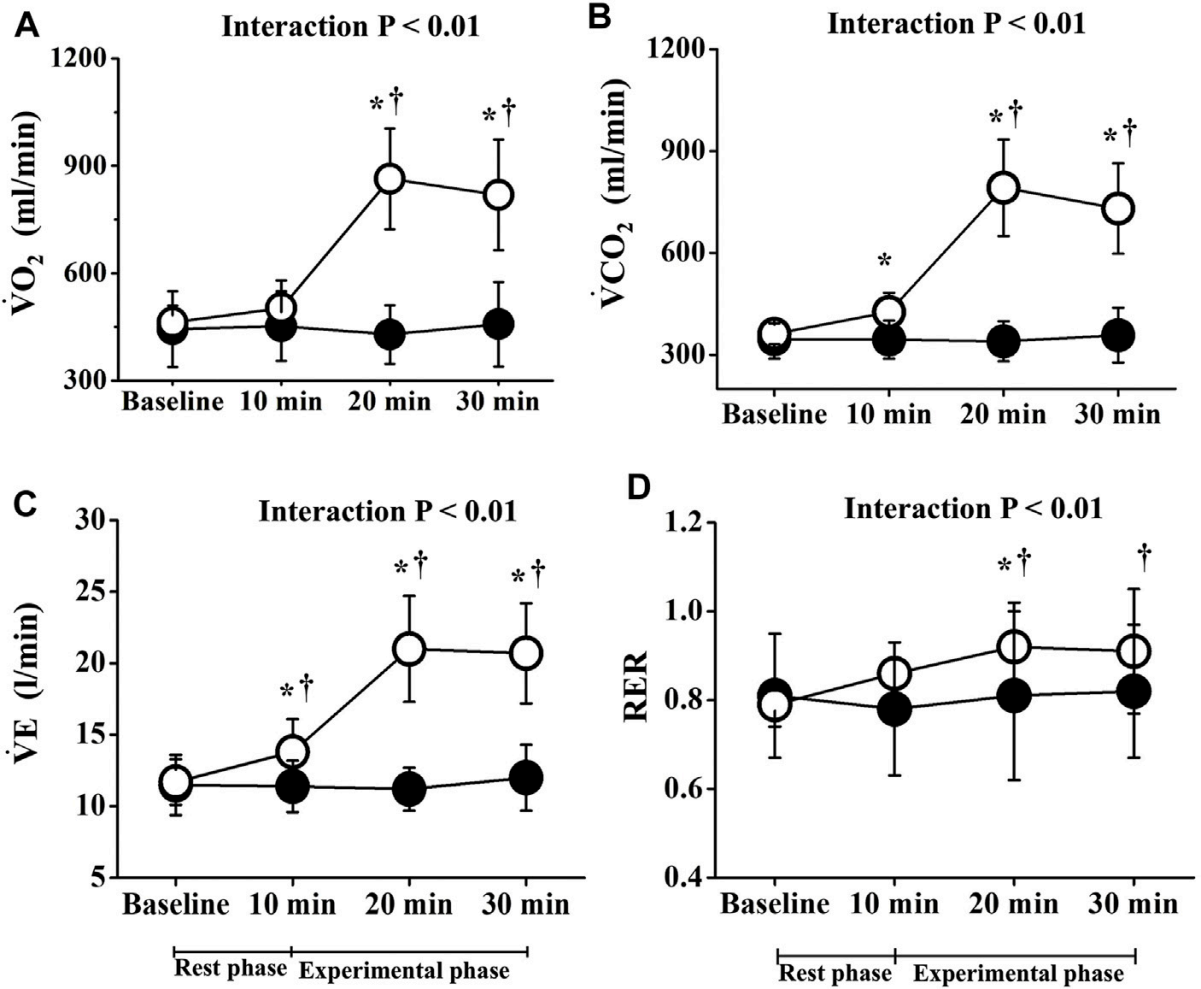
Temporal changes in respiratory parameters. Filled circles (•), control resting trial; open circles (○), electrical stimulation trial; ${\text{V}}^{・}$ O_2_, oxygen uptake; ${\text{V}}^{・}$ CO_2_, carbon dioxide output; ${\text{V}}^{・}$ E, minute expired ventilation; RER, respiratory exchange ratio; Baseline, before each trial. Other abbreviations are the same as in Figure 1. ∗*p* < 0.01 vs. CT; †*p* < 0.01 vs. baseline. Data are given as mean ± SD.

Two-way repeated-measures ANOVA revealed significant interactions in PR, lactate, and respiratory gas parameters, respectively (*p* < 0.01; Figures 2, 3). No significant differences in these baseline parameters were observed between both trials, and parameters of the CT did not change significantly throughout the study period. Conversely, PR in the ET increased significantly from the baseline value during EMS and was significantly higher than that in CT. Mean differences between resting baseline and during EMS and between both trials were 14 ± 5 beats/min and 15 ± 11 beats/min, respectively. Circulating lactate levels in the ET were significantly increased only at Post 1. During EMS, each respiratory gas parameter significantly increased from baseline levels and values were higher than those of CT, as well as PR.

### Effects of EMS on Arterial Stiffness and Hemodynamic Parameters

**FIGURE 4:**
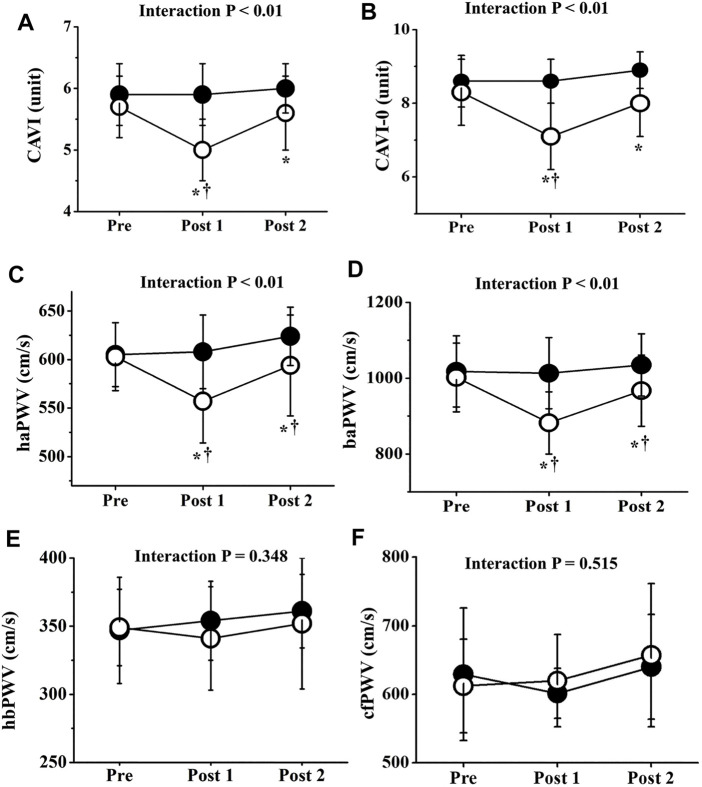
Temporal changes in arterial stiffness parameters. CAVI **(A)**, CAVI0 **(B)**, haPWV **(C)**, baPWV **(D)**, hbPWV **(E)**, and cfPWV **(F)** are shown. Filled circles (•), control resting trial; open circles (○), electrical stimulation trial; CAVI, cardio-ankle vascular index; CAVI0, cardio-ankle vascular index 0; haPWV, heart-ankle pulse wave velocity; baPWV, brachial-ankle pulse wave velocity; hbPWV, heart-brachial pulse wave velocity; cfPWV, carotid-femoral pulse wave velocity. Other abbreviations are the same as in Figure 1. ∗*p* < 0.01 vs. CT; †*p* < 0.01 vs. Pre. Data are given as mean ± SD.

**FIGURE 5:**
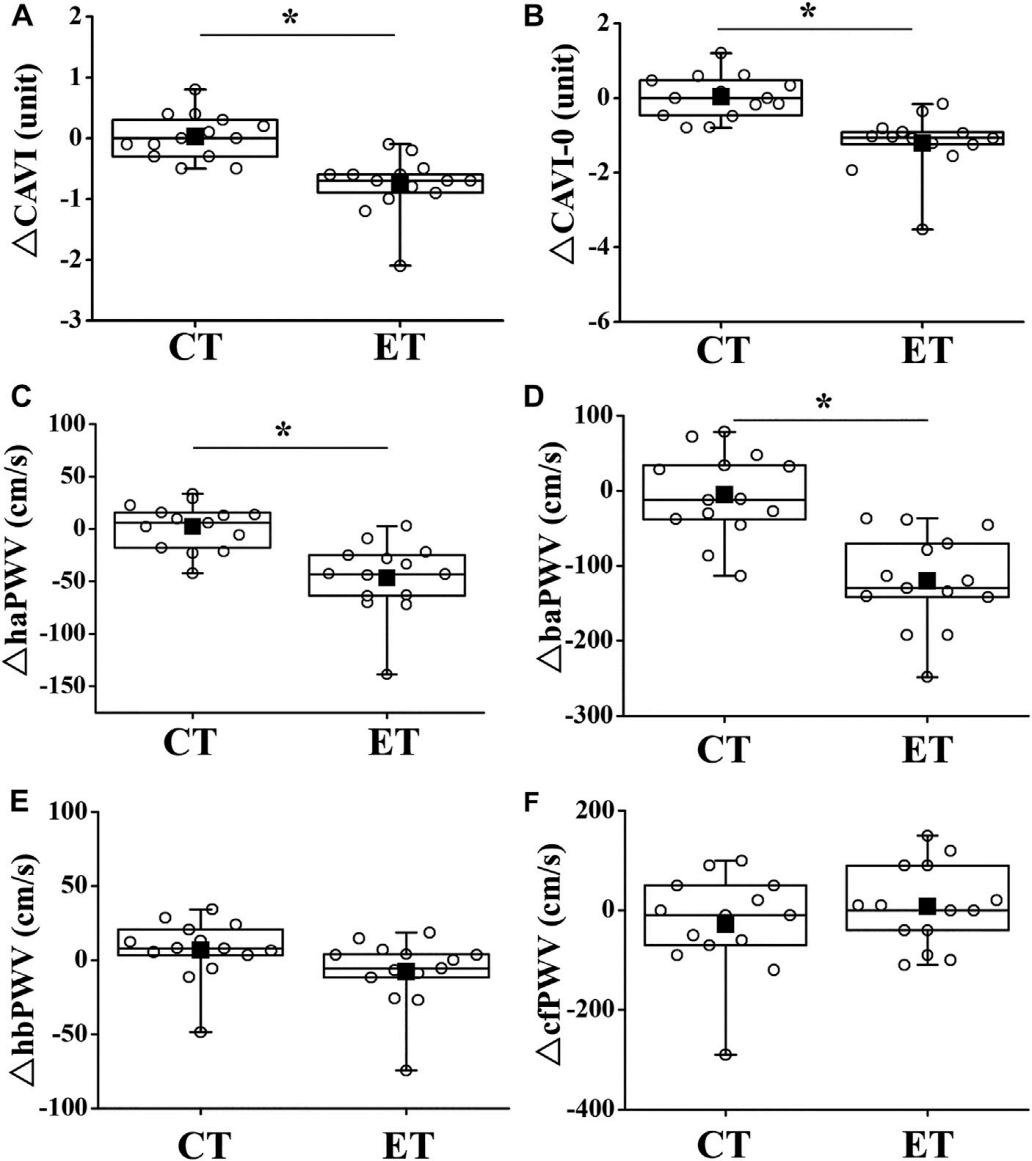
Comparisons of change in arterial stiffness between CT and ET. Box-and-whisker plots indicate that the horizontal bar within the box denotes the median value. The top and bottom borders of the box mark the 75th and 25th percentiles. The ends of the whiskers are drawn to the minimum and maximum points. Filled squares (■) and open circles (○) show mean value and personal values, respectively. CT, control resting trial; ET, electrical stimulation trial. Abbreviations for arterial stiffness parameters are the same as in Figure 4. ∗*p* < 0.01 vs. CT.

Two-way repeated-measures ANOVA revealed significant interactions in CAVI, CAVI_0_, haPWV, and baPWV (*p* < 0.01), but not in hbPWV or cfPWV (Figure 4). CAVIs and PWVs at Pre did not differ significantly between trials, and CAVIs and PWVs in the CT did not change significantly throughout the study period. On the other hand, in the ET, CAVI (Post 1, ES = 1.40; Post 2, ES = 0.18), CAVI_0_ (Post 1, ES = 1.55; Post 2, ES = 0.96), haPWV (Post 1, ES = 1.16; Post 2, ES = 0.19), and baPWV (Post 1, ES = 1.38; Post 2, ES = 0.38) were all significantly reduced at both Post 1 and Post 2 (*p* < 0.01). CAVI, CAVI_0_, haPWV, and baPWV values at Post 1 were also significantly lower in the ET than in the CT. Furthermore, value of reductions in CAVI, CAVI_0_, haPWV, and baPWV were significantly greater in the ET than in the CT (*p* < 0.01, Figure 5). However, hbPWV and cfPWV did not change significantly after the ET.

**FIGURE 6:**
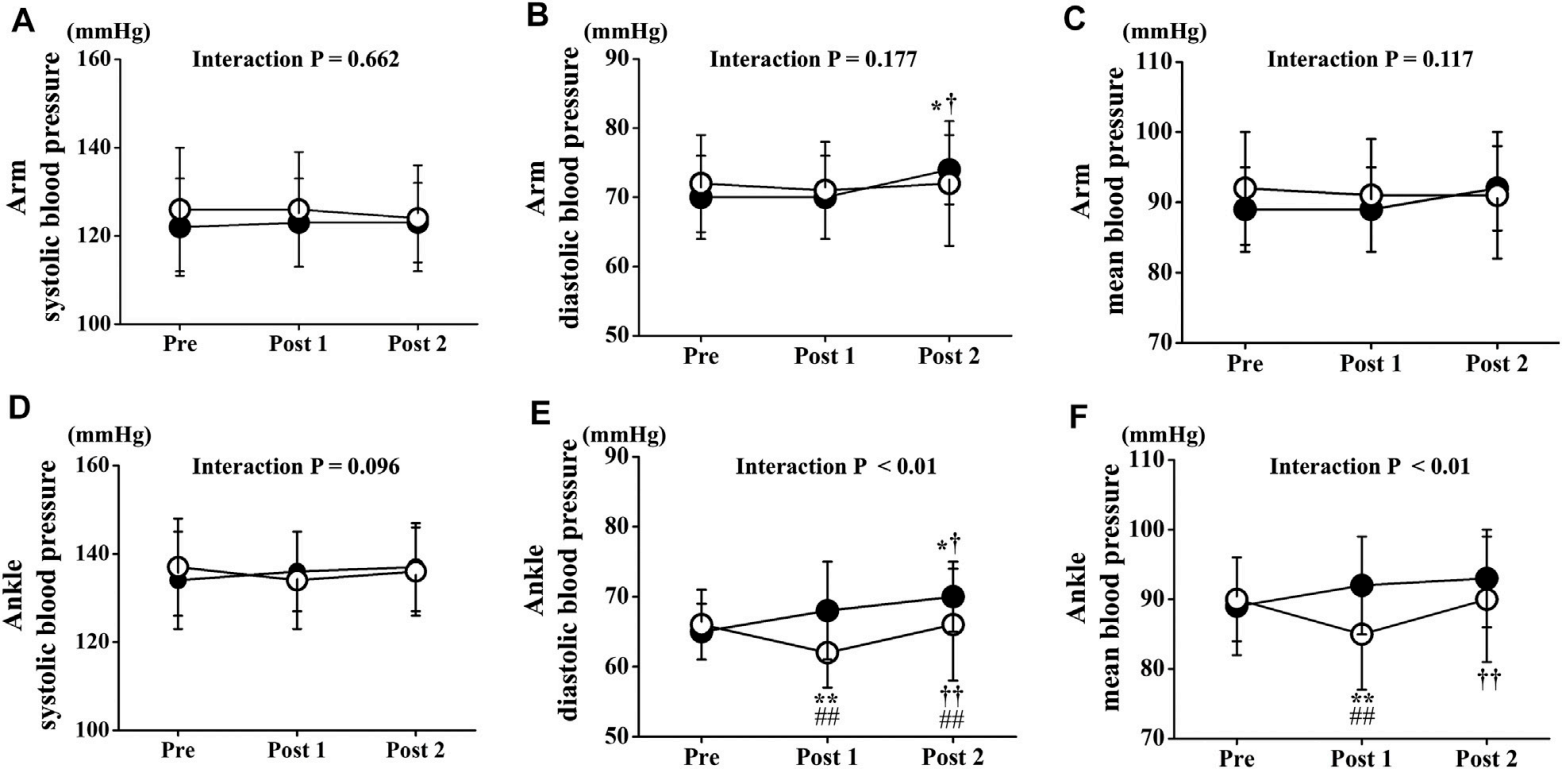
Temporal changes in arm and ankle blood pressure. Arm systolic **(A)**, diastolic **(B)**, and mean **(C)** blood pressure and ankle systolic **(D)**, diastolic **(E)**, and mean **(F)** blood pressure are drawn. Filled circles (•), control resting trial; open circles (○), electrical stimulation trial; Data are expressed as mean ± SD. ∗*p* < 0.05: vs. Pre, ∗∗*p* < 0.01: vs. Pre, †*p* < 0.05: vs. Post 1, ††*p* < 0.01: vs. Post 1, #*p* < 0.05: vs. CT, ##*p* < 0.01: vs. CT.

Two-way repeated-measures ANOVA revealed no significant interactions in arm BPs. Arm BPs at Pre did not differ significantly between both trials. Arm systolic BP and mean arm BP did not change significantly in either trial throughout the experimental period. However, arm diastolic BP at Post 2 was slightly but significantly increased compared with Pre or Post 1 in the CT, but not in the ET (*p* < 0.05 Figure 6B). Conversely, two-way repeated-measures ANOVA revealed significant interactions in ankle BPs (diastolic BP: *p* < 0.01, mean BP: *p* < 0.01, Figures 6E,F). Ankle BP at Pre did not differ significantly between both trials, and ankle systolic BP in the both trials did not change significantly throughout the study period. However, ankle diastolic and mean BP at Post 1 was significantly lower in the ET than in the CT (diastolic BP: *p* < 0.01, mean BP: *p* < 0.01). Ankle diastolic BP at Post 2 was also significantly lower in the ET than in the CT (*p* < 0.05). Ankle diastolic and mean BP were significantly reduced between Pre and Post 1 in the ET (*p* < 0.01). Ankle diastolic BP at Post 2 was significantly increased compared with Pre or Post one in both trials (CT: vs. Pre *p* < 0.05, vs. Post 1 *p* < 0.05, ET: vs. Post 1 *p* < 0.01).

## Discussion

The salient findings from this investigation were that low-intensity EMS of the lower limbs significantly reduced CAVI, CAVI_0_, haPWV, and baPWV, but not cfPWV or hbPWV. These findings suggest that acute EMS reduces arterial stiffness and that this reduction in arterial stiffness is not uniformly observed among the segments.

In the present study, the intensity of EMS was set a 15 ± 7 beats/min increase in PR compared to the baseline value, and mean levels during EMS were 77 ± 6 beats/min (10.7 ± 4.7% HRR), which indicates light-intensity exercise stimulus (Garber et al., 2011). Respiratory gas parameters in the ET also increased significantly during EMS, and the intensity of energy metabolism corresponded to approximately 2 METs (1.8 ± 0.3), the same level as standing (Ainsworth et al., 2000). Although lactate levels increased slightly after EMS, the mean value was 2.2 ± 0.6 mmol/L, which did not reach the threshold for onset of blood lactate accumulation (OBLA). Therefore, in terms of physiological indicators during EMS, the intensity of our EMS of the lower limbs was similar to or lower than walking, indicating that our EMS was low intensity.

Interestingly, CAVI, CAVI_0_, haPWV, and baPWV were significantly reduced by EMS, whereas hbPWV and cfPWV were not. BP itself is well known to affect PWV values (Benetos et al., 1993), and arm BP did not significantly alter throughout the ET. However, ankle diastolic and mean BPs were decreased by EMS. Furthermore, CAVI is generally an indicator of systemic PWV (i.e., haPWV) after adjusting for BP, and haPWV comprises hbPWV and baPWV elements (Kume et al., 2020; Ogawa et al., 2020). In the present study, no significant changes in hbPWV were observed, reflecting arterial stiffness of the upper limbs from the aorta to the brachium (Sugawara et al., 2019). Conversely, baPWV in the ET was significantly reduced after EMS, which is explained by PWV elements of the central parts (large arteries in the cardiothoracic region) and lower limbs from the femur to the ankle (Sugawara et al., 2005). Our data also indicate that cfPWV, which is generally proposed as the reference standard for central arterial stiffness, did not change significantly after EMS. The reductions in CAVI, CAVI_0_, haPWV, and baPWV therefore strongly suggest a reduction in arterial wall stiffness, especially in lower limbs associated with areas that received EMS. In other words, low-intensity EMS of the lower limbs did not appear to affect arterial stiffness of the central and upper limbs, revealing site differences.

Increased blood flow is considered to be one of the key factors contributing to acute exercise-induced reductions in arterial stiffness. Vascular smooth muscle tone is affected by vasoactive substances such as nitric oxide (NO) synthesized by vascular endothelial cells. Previous *in vivo* studies have shown that NO reduces arterial stiffness (Kinlay et al., 2001). In addition, endothelial NO synthase (eNOS) is associated with increased production of NO due to EMS (Balon and Nadler, 1994). The production of NO, as a potent endothelial dependent vasodilator that also reduces vasoconstrictor response to α-adrenergic receptor stimulation, is enhanced with increased blood flow and cyclic shear stress associated with pulsatile blood flow and acute exercise (Patil et al., 1993; Jungersten et al., 1997). The muscle contraction due to EMS causes increases in shear stress and blood flow (Hudlicka, 1998; Janssen and Hopman, 2003). In the present study, PR was increased by about 15 beats/min by EMS, so EMS may have increased blood flow, as previously suggested. Our results indicated that ankle BP was significantly reduced after EMS, and the alteration in ankle BP may affect the reduction in arterial stiffness in lower limb. Thus, we calculated CAVI0 using the value of ankle BP, and then confirmed that the values and changes of CAVI0 are approximately the same results between ankle BP and arm BP (Interaction: *p* < 0.01, Post 1, ES = 1.39; Post 2, ES = 0.17). The decrease in arterial stiffness with low-intensity EMS is therefore considered to be influenced by the increase in NO production due to increased blood flow. From the above, a potential mechanism explaining the acute reduction in segmental arterial stiffness may be related to increased segmental blood flow due to passive muscle contraction by EMS.

We can only speculate on the physiological mechanisms by which EMS reduces arterial stiffness only in sites that received EMS. A study has reported that acute exercise can induce a greater reduction in peripheral parts than in central parts (Kingwell et al., 1997), and cfPWV, as an index of central arterial stiffness, is considered to be hard to reduce after acute aerobic exercise. Moreover, the result of site difference may be influenced by the lack of increase in blood flow to the non-EMS limb (especially from heart to brachial). Sugawara et al. showed that the PWV from femoral to ankle arteries in exercised legs was significantly decreased 5 min after low-intensity, single-leg exercise (an approximately 30 beats/min increase in heart rate from baseline), while the non-exercised leg did not show any change (Sugawara et al., 2003). They therefore concluded that the lack of reduction in arterial stiffness in non-exercised limbs was due to the lack of increase in blood flow to the non-exercised limbs as a result of the low intensity of exercise. Tanaka et al. reported that low-intensity leg cycle exercise did not increase brachial blood flow or shear stress (Tanaka et al., 2006). Tanaka et al. also reported that brachial blood flow and shear stress did not differ from resting level when the heart rate was approximately 80 beats/min for leg cycle exercise (Tanaka et al., 2006). EMS in this study was applied at low intensity, as a 15 ± 5 beat/min elevation in PR (to 77 ± 6 beats/min), so blood flow may not have been increased at non-EMS sites such as the brachial artery. Thus, it is possible that the arterial stiffness of non-EMS site did not reduce because of the lack of blood flow increases. In the future, measurements of regional blood flow during acute low-intensity EMS are needed to assess this hypothesis.

Regular aerobic exercise (e.g., walking, cycling, and swimming) is important to reduce arterial stiffness. A recent study reported that acute low-intensity aerobic exercise (30–50% HRR) reduced arterial stiffness (an approximately −15% reduction in CAVI) in young people (Wang et al., 2014; Zhou et al., 2015). The present data showed that acute low-intensity ES produced a −13.1 ± 7.8% and −14.4 ± 8.4% reduction in CAVI and CAVI_0_ as an index of arterial stiffness, although intensity of EMS in the present study was 10.7 ± 4.7% of HRR. Moreover, METs during EMS corresponded to approximately 2 METs (1.8 ± 0.3), comparable to that during standing. The intensity of EMS was thus very low, but low-intensity EMS may generally induce similar reductions in arterial stiffness compared with acute low-intensity regular aerobic exercise. Interestingly, arterial stiffness was reduced despite the low-intensity EMS. This reduction in arterial stiffness due to EMS appears to be affected by increased local blood flow due to passive muscle contraction, and this response was only in sites that received EMS. However, such EMS would still be beneficial for individuals with lower limb disease. In addition, adding other exercise methods (e.g., arm cranking) may be an effective method. We therefore believe that these data represent useful information for the development of effective intervention programs using EMS.

Increased arterial stiffness is widely accepted as an independent risk factor for future cardiovascular diseases or mortality (Laurent and Boutouyrie, 2007; Vlachopoulos et al., 2010; Vlachopoulos et al., 2012). Recent studies report that long-term EMS intervention reduces arterial stiffness in patient with pulmonary arterial hypertension and chronic heart failure and has a therapeutic effect on hypertension (Dobsak et al., 2012; Kahraman et al., 2020). The present data indicate that acute low-intensity EMS produced a reduction in arterial stiffness in young subjects, as supported by previous studies. However, the effects of EMS were local, and further investigations are thus required to clear the methods for reducing central arterial stiffness by low-intensity EMS.

Finally, several important limitations need to be considered for this study. We calculated appropriate sample sizes before the study, but the cohort of participants was homogeneous, comprising healthy young individuals. In general, large elastic arteries progressively stiffen with age (Avolio et al., 1985; Vaitkevicius et al., 1993; Nishiwaki et al., 2014). Therefore, additional interventions for elderly subjects may reveal important new insights into arterial stiffness alterations and adaptations by EMS. Although our pilot data indicated that 4 weeks of EMS intervention reduced arterial stiffness even in young male participants, further large-scale investigations are required to determine whether our findings can be extrapolated to other populations.

## Conclusion

Results from the present study demonstrated that low-intensity EMS of the lower limbs reduced arterial stiffness. Moreover, arterial stiffness was only reduced in sites at which EMS was performed.

## Data Availability

The raw data supporting the conclusion of this article will be made available by the authors, without undue reservation.
